# Medical assessment in the age of digitalisation

**DOI:** 10.1186/s12909-020-02014-7

**Published:** 2020-03-31

**Authors:** Saskia Egarter, Anna Mutschler, Ara Tekian, John Norcini, Konstantin Brass

**Affiliations:** 1 Institute for Communication and Assessment Research, Wieblinger Weg 92A, Heidelberg, Germany; 2 Center of Excellence Assessment in Medicine, University of Heidelberg, Heidelberg, Germany; 3 Department of Medical Education, University of Illinois, Chicago, USA; 4 Foundation for the Advancement of International Medical Education Research, Philadelphia, PA USA

**Keywords:** Digital assessment, Medical education, ItemManagementSystem, Electronic exams, Item design, Assessment Alliance

## Abstract

**Background:**

Digital assessment is becoming more and more popular within medical education. To analyse the dimensions of this digital trend, we investigated how exam questions (items) are created and designed for use in digital medical assessments in Germany. Thus, we want to explore whether different types of media are used for item creation and if a digital trend in medical assessment can be observed.

**Methods:**

In a cross-sectional descriptive study, we examined data of 30 German medical faculties stored within a common assessment platform. More precise, 23,008 exams which contained 847,137 items were analysed concerning the exam type (paper-, computer- or tablet-based) and their respective media content (picture, video and/or audio). Out of these, 5252 electronic exams with 12,214 questions were evaluated. The media types per individual question were quantified.

**Results:**

The amount of computer- and tablet-based exams were rapidly increasing from 2012 until 2018. Computer- and tablet-based written exams showed with 45 and 66% a higher percentage of exams containing media in comparison to paper-based exams (33%). Analysis on the level of individual questions showed that 90.8% of questions had one single picture. The remaining questions contained either more than one picture (2.9%), video (2.7%), audio (0.2%) or 3.3% of questions had picture as well as video added. The main question types used for items with one picture are TypeA (54%) and Long_Menu (31%). In contrast, questions with video content contain only 11% TypeA questions, whereas Long_Menu is represented by 66%. Nearly all questions containing both picture and video are Long_Menu questions.

**Conclusions:**

It can be stated that digital assessment formats are indeed on the raise. Moreover, our data indicates that electronic assessments formats have easier options to embed media items and thus show a higher frequency of media addition. We even identified the usage of different media types in the same question and this innovative item design could be a useful feature for the creation of medical assessments. Moreover, the choice of media type seems to depend on the respective question type.

## Background

Artificial intelligence, big data, university 4.0, digital transformation and digitalisation are current trends and buzzwords highly discussed in the world of health care and higher education [1–3]. Especially the topic of digitalisation is reaching an omnipresence within society and meanwhile takes on an important role within higher education. Digitalisation not only describes the transition from analogous to digital data, but also refers to the consequences that occur due to this digital shift. Computers, tablets and smartphones are capable to process data digitally and are nowadays inevitable electronic devices in health care, medicine and medical education [4–6]. These digital media will sooner or later change the way we think of and handle daily and critical health care situations [7]. On the basis of these facts, we observe new tasks in medical education which address the teaching and assessment of digital competencies of medical examinees, implying the necessity to define these competencies [8, 9].

Digitalisation encompasses the emergence of novel tools and applications which offer many new opportunities within the educational sector, especially concerning new assessment formats and content of exams [10]. For this reason, technical aspects have to be revised and optimised for the implementation of electronic assessments within medical education [8]. The benefits of these electronic assessments are on the one hand faster correction of exams through automated evaluations. In addition, E-Assessment in general is less prone to errors and thus leads to an increased quality of the exam [11]. On the other hand, the availability of numerous digital assessment tools changes the process of exam creation and new assessments formats can be applied. Modern technologies offer new possibilities to create items with innovative designs [12]. Due to these new technological possibilities, audio-visual media content can be added to support the textual structure of exam questions. Moreover, multiple media types can be combined in the same exam question leading to an advanced question composition.

The potential addition of media content to questions is the benefit of E-Assessment. In the context of medical education, a study area with the most performed electronic exams [13], electronic exams have the advantage that high resolution images of diseases or medical conditions can be illustrated without losing quality due to printing on paper. Furthermore, digital image marking is another asset of digital assessments. This item type can be used to mark a specific point in an image like a foreign object in a lung (called Hot Spot [12]).

The usage of digital tools on mobile devices allows a layered and dynamic item design, and thus the medical educator has become a designer of items in assessments [14]. The constructed environment (real or virtual) offers new opportunities to act [15]. Therefore, it is essential to examine the already ongoing process of digitalisation in medical schools [16]. It is unavoidable that the use of computer assisted systems and mobile devices in medical education will change how we will assess medical knowledge in the future [17]. In the age of digitalisation more and more electronic assessments are performed in medical education in Germany [18]. Hence, this article focuses on the “digital” status quo of assessments in German medical schools and discusses exams in the context of their respective exam type and media content.

## Methods

### Data acquisition and ItemManagementSystem

The basis for our data evaluation is the assessment platform ItemManagementSystem (IMS) of the Umbrella Consortium for Assessment Networks (UCAN) [19, 20], where data of roughly 35,042 performed exams with 616.543 unique items from 70 institutions are stored. For this cross-sectional descriptive study, we focused on data from 30 German medical faculties which stored in total 28,376 exams that included 957,059 questions (several questions were used in more than one exam). After confining the exams to the selected time frame from 2012 to 2018, 26,742 exams with 856,150 questions remained. This time frame was chosen because from 2012 media elements were used in electronic exams. Since we cannot control whether the users of the IMS provide correct information about their exams, we filtered the exams and excluded exams with less than 5 questions and exams having the word “test” in their description with less than 10 questions. This represents the best possible way to exclude most exams generated to test the system or new features of the IMS. After disregarding above mentioned exams, 23,008 exams that contained 847,137 items were evaluated regarding their exam type (paper, computer and tablet) and included media content (pictures, videos or audio).

For the investigation of the media usage in exam questions, 17,756 paper-based exams were disregarded because paper-based exams offer less possibilities for media addition compared to electronic exams. Thus, the remaining 5252 computer- and tablet-based exams with 205,988 questions were further analysed. For tablet-based exams we distinguish between tablet-based written assessments (tEXAM) and Objective Structured Clinical Examinations (OSCE) evaluated by the examiners via tablet (tOSCE). tOSCE was introduced in 2013 and tEXAM in 2015.

First, the different media types per single question were quantified and second the preferred question type for each media type determined. We categorised the questions into six different classes: TypeA, Pick-N, Kprime, Freetext, Long_Menu and OSCE:

- TypeA questions are multiple-choice questions with one correct answer.
- Pick-N questions are also multiple-choice questions, but with more than one correct possible answer.
- Kprime questions are basically yes or no questions where each possible answer must be marked as true or not true.
- Freetext questions have a textbox where any desired answer can be given.
- Long_Menu question have usually a long answer list (e.g. 10,000 diagnoses) to choose from and thus are comparable to freetext questions where the knowledge of the right answer is presumed.
- OSCE is an assessment format for skill testing in written or tablet-based form that was first described by Harden et al. [21, 22]. In our case, tablet-based OSCE refers to the evaluation mode of the OSCE which is performed via a tablet. OSCE questions refer to questions used in OSCE assessments. These questions can be a mixture of any of the above-mentioned question types.

### Software and statistical analyses

The evaluation of the compiled data occurred through the software packages Microsoft Excel (Microsoft Office version 2019, Microsoft Corporation, Redmond WA, USA). With the Excel software mean values and standard deviations were determined. The dependency of question type and media type was analysed with R (version 3.4.3, R Core Team) using Pearson’s Chi-squared test.

## Results

### Different exam types and tendencies in electronic assessment

**Fig. 1:**
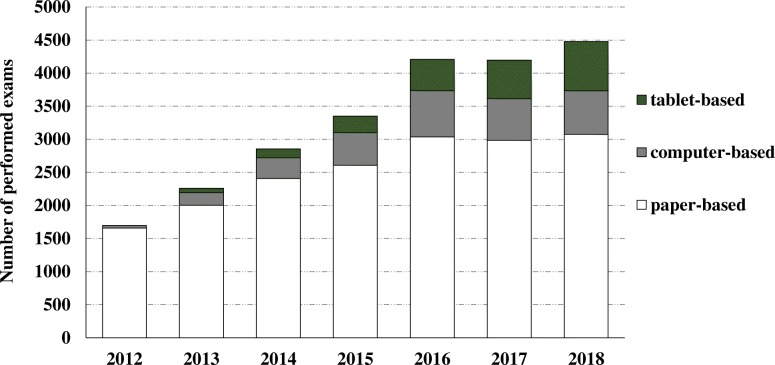
Performed exams categorised by exam type between 2012 and 2018

Figure 1 illustrates the number of performed exams categorised into three different exam types, and shows the frequent usage of paper-based exams which were rapidly increasing from 2012 until 2015 where they seem to stay stable with around 3000 exams per year. In the year 2010 the first computer-based exams were performed (not shown in graph) and akin to paper-based exam, the numbers were increasing every year. A similar effect can be observed for tablet-based assessments, an exam type that was introduced 2013 and proved quite popular, with a higher number of performed exams in 2018 compared to other electronic assessment types.

### Item design and use of items containing media

**Fig. 2:**
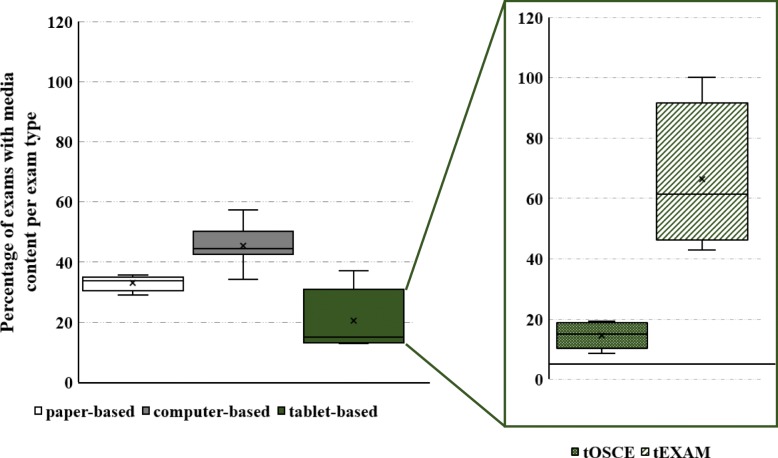
Percentage of exams with media content per exam type

The average percentage of exams containing media with reference to all exams of the respective exams type is illustrated in Fig. 2. While 33% of all paper-based exams contain a media element, 45% of computer-based exams and 20% of tablet-based assessments have media added. OSCEs assess the practical clinical skills of medical examinees and thus it is probably not surprising that quite few media items (14,5%) are added to these exams. On the other hand, tEXAM assessments contain on average 66% media, which is double the amount compared to paper-based exams.

**Fig. 3:**
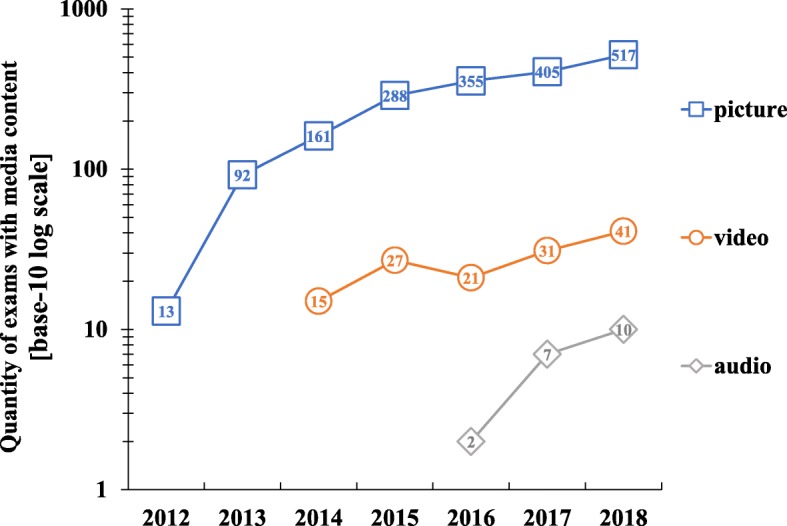
Quantity of exams with media content in electronic assessment per year are illustrated on a base-10 log scale on the Y axis (N = total number of exams with media content)

In the investigations what kind of media types are used in electronic exams, it is not surprising that pictures are still the main media type used in combination with text (Fig. 3). Additionally, the quantity of exams with picture content is increasing every year and most likely will further increase in the future. The second most used media type are videos, which were added to exams for the first time in 2014. Their numbers seem, compared to exams with pictures, quite stable with approximately 30–40 performed exams per year (Fig. 3). The last media type utilised are audio data attached to questions. This media type is only used for 3 years and the numbers are still quite low with ten exams in 2018, but a tendency to increase in the future can be observed (Fig. 3).

### Allocation of media during exam question creation

**Fig. 4:**
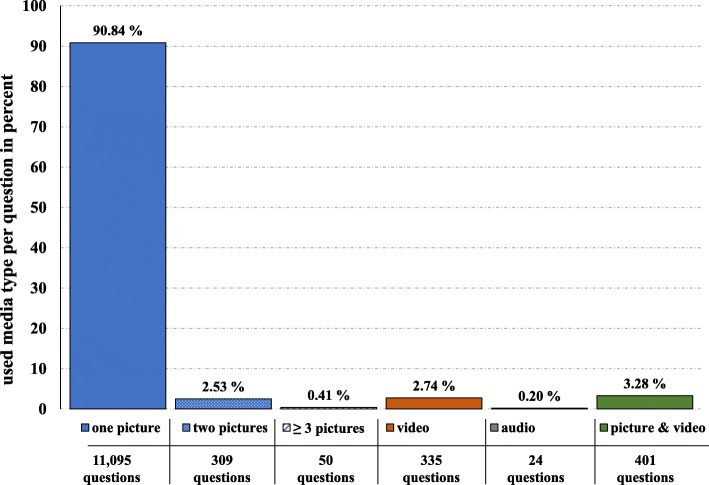
Media usage in electronic exam questions

To analyse the used media type in E-Assessment, 5252 electronic exams were evaluated with 2065 exams that contained either picture, video or audio as media types. These exams contained 12,214 questions that were further assessed individually regarding their added media types. In accordance with the above-mentioned data, the evaluation of questions showed that pictures are indeed the main media type in combination with the question text (Fig. 4). While 11,095 questions (90.8%) contained one picture, 1119 questions had other media types. Out of these 1119 questions, 309 questions (2.5%) contained two pictures per question, and 50 questions (0.4%) had three or more pictures added. Few questions had even 5 pictures attached to it. Apart from pictures, 335 questions (2.7%) contained videos and 24 questions audios (0.2%). Furthermore, we observed 401 questions (3.3%) that had both picture as well as video additions (Fig. 4), which represents a feature only possible in digital assessment.

**Fig. 5:**
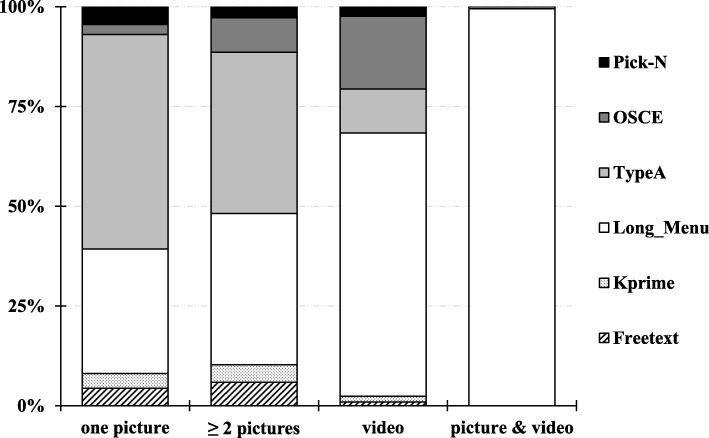
Usage of different media types categorised by question type

We investigated whether a particular media type is favoured for a special question type. Our findings show that 54% of the questions containing one picture can be assigned to single answer multiple choice questions (TypeA) (Fig. 5) and 31% of the questions belong to the question type Long_Menu. The question types Pick-N, OSCE, Kprime and Freetext are represented with less than 5% each. An analogous result was observed for questions with 2 and more pictures, whereby Long_Menu and OSCE questions slightly increased to 37 and 9% respectively. Surprisingly, questions containing videos show a completely different pattern. Single answer multiple choice questions, for instance, cover only 11% of all questions with video data, whereas most of this media type is applied to Long_Menu questions (66%). OSCE questions containing video are represented with 18%. Finally, we analysed the second most common type of media, the use of both image and video, and found that they occur mainly in Long_Menu questions. However, it has to be noted that the combination of these two media types in exam questions is only used by two of the evaluated institutions. The execution of Pearson’s Chi-squared test showed a dependence of question type and media type (X -squared = 1344.3, df = 15, *p*-value < 2.2e-16).

## Discussion

The current study shows the digital trend towards electronic exams in German medical schools. The quantity of electronic assessment methods, such as computer- and tablet-based exams has grown strongly until the end of 2018 according to our analysis. The frequent usage of picture items could be demonstrated with our data since pictures still represent the main media type used in medical assessments. Our data shows that other media types such as audio and video are quite rarely used within medical education in Germany. Perhaps these media types are more advantageous for other areas of studies. Within the musicology, parts of musical works could be played and learning foreign languages native speakers could be listened to which is already an element of learning management systems [23–25] and part of E-learning platforms used to learn foreign languages [26, 27].

In general, good quality questions used for exams are costly [28] and one of the drawbacks of video data is that the production and reuse in following exams might be expensive. For this reason, assessment platforms that are shared by multiple users open new possibilities and can be applied to solve this issue. In the course of digitalisation, the number of digital tools for medical assessment is increasing rapidly and numerous tools for the use of media are available. Videos can be captured and uploaded instantly. Following this, the videos can even be annotated during an exam. Thus, we speculate that the use of various media in exam questions will become more and more popular in the future. Other drawbacks of using audio and video files include data protection rights and copyrights, especially when data resources of patients are intended to be used. Moreover, video and audio files are more prone to technical issues compared to pictures.

Unfortunately, the authors did not find any references regarding nationwide studies to determine a change in media usage after implementation of digital exams. However, there is a trend in the use of digital assessment formats worldwide [29–32]. As early as 2004, the British government announced a very ambitious project to expand the use of E-Assessment [33]. They state that “E-assessment can take a number of forms, including automating administrative procedures; digitising paper-based systems, and online testing” indicating that digitalisation of medical assessments occurs on several levels apart from the exam delivery mode itself. Many studies are available, that report the transition of traditionally pen and paper testing to electronic exam [32, 34–37]. This change leads to an increase of electronic exam delivery and can be found worldwide and in several areas of study and is in accordance with our findings, that the number of electronic exams is continuously increasing.

There are some limitations to our study. First, we only analysed the data of one specific database which depends on the accurate entering of the data by its users. Second, we have only focused on medicine as a subject of study and only considered German medical faculties. Third, our exam exclusion criteria may have resulted in exclusion of potentially relevant exams or questions. Moreover, the number of some question type and media type combination in our analysis might be rather low for less frequently used question types. Nonetheless, this study is the first attempt to compare and analyse the used media types for medical exam questions in different exam types.

Further research could incorporate the analysis whether the use of media in exam questions is beneficial for the quality of the assessments and thus the quality of medical study. For that reason, statistical data like the difficulty, discriminatory power and reliability of exam questions with and without media should be collected. An automatic upload of statistical values does currently not take place in the examined assessment platform, but is planned for the current year, which is why a statistical analysis of exam questions would be feasible in the future and is aimed at. Furthermore, it should also be explored to what extent different medical competencies and knowledge can be tested depending on the chosen media and question type. Hurtubise and colleagues discussed the usage of video clips linked to different competency domains [38]. In Germany, medical competencies are defined, among others, in the National Competence Based Catalogue of Learning Objectives for Undergraduate Medical Education (NKLM; Nationaler Kompetenzbasierter Lernzielkatalog in der Medizin) which serves as an orientation for the medical faculties and has the character of a recommendation [39]. The professional roles taken by physicians are derived from the Canadian CanMEDS framework concept, which originally referred to a level of competence in specialist medicine, but which has gained wide international acceptance and dissemination for medical training [40]. The model was transferred to the competence level of the NKLM. It would be conceivable to explore the use of media for the various areas of competence of the NKLM or the various CanMEDS roles, and to determine whether certain types of media are used more frequently for certain areas of competence or roles.

## Conclusion

In the age of digitalisation, a digital trend is occurring in higher education in Germany. In our study we analysed how this digital change is affecting assessments in medical education. Interestingly, an increased number of media additions like picture, video and audio can be observed in electronic exams and even media combinations of text, picture and video are utilised for the creation of exam questions. So far, the usage of different media types in the same question is quite rare, but modern digital technologies together with the collective exchange of items in a common item-pool and a broad range of available assessment tools will facilitate these innovative digital assessment formats in the future. In addition, our study demonstrates that some media types are more used for a specific question type than others. Thus, one could speculate that our data shows that specific media types are more suitable for some question types than for others.

## Data Availability

The datasets used and/or analysed during the current study are available from the corresponding author on reasonable request.

## Abbreviations

CanMEDS: Canadian Medical Education Directives for Specialists
E-Assessment: Electronic Assessment
E-learning platform: Electronic learning platform
IMS: ItemManagementSystem
NKLM: Nationaler Kompetenzbasierter Lernzielkatalog in der Medizin (National Competence Based Catalogue of Learning Objectives for Undergraduate Medical Education)
OSCE: Objective Structured Clinical Examinations
tEXAM: Tablet-based written exams
tOSCE: Tablet-evaluated OSCE
UCAN: Umbrella Consortium for Assessment Networks
